# Multiplex parenting: IVG and the generations to come

**DOI:** 10.1136/medethics-2013-101810

**Published:** 2014-03-07

**Authors:** César Palacios-González, John Harris, Giuseppe Testa

**Affiliations:** 1 Institute for Science Ethics and Innovation, The University of Manchester, Manchester, UK; 2 Laboratory of Stem Cell Epigenetics and Research Unit on Biomedical Humanities, European Institute of Oncology, Milan, Italy

**Keywords:** artificial gametes, synthetic gametes, multiplex parenting, in vitro produced gametes, stem-cell derived gametes

## Abstract

Recent breakthroughs in stem cell differentiation and reprogramming suggest that functional human gametes could soon be created in vitro. While the ethical debate on the uses of in vitro generated gametes (IVG) was originally constrained by the fact that they could be derived only from embryonic stem cell lines, the advent of somatic cell reprogramming, with the possibility to easily derive human induced pluripotent stem cells from any individual, affords now a major leap in the feasibility of IVG derivation and in the scope of their potential applications. In this paper we develop an ethical framework, rooted in recent scientific evidence, to support a robust experimental pipeline that could enable the first-in-human use of IVG. We then apply this framework to the following objectives: (1) a clarification of the genetic parenting options afforded by IVG, along with their ethical underpinnings; (2) a defence of the use of IVG to remedy infertility, broadening their scope to same-sex couples; (3) an assessment of the most far-reaching implications of IVG for multiplex parenting. These include, first, the liberation of parenting roles from the constraints of biological generations in vivo, allowing multiple individuals to engage in genetic parenting together, thus blurring the distinction between biological and social generations. Second, we discuss the conflation of IVG with sequencing technology and its implications for the possibility that prospective parents may choose among a hitherto unprecedented number of potential children. In view of these perspectives, we argue that, contrary to the exhausted paradigm according to which society lags behind science, IVG may represent instead a salient and most visible instance where biotechnological ingenuity could be used in pursuit of social experimentation.

## Introduction

Nearly 10 years have elapsed since two of us pioneered the discussion of the ethical implications of in vitro generated gametes (IVG).^1^ ^2^ Since then the ethical discussion regarding the possible uses of such gametes has attracted considerable attention in the bioethics literature.^3–9^ Our discussion of the ethical implications of IVG was undertaken at a time when these could only be derived from existing lines of human embryonic stem cells (hESCs), obtained from in vitro fertilisation (IVF) or somatic cell nuclear transfer embryos, a context that severely limited their potential.^1^ ^2^ The advent of somatic cell reprogramming, and with it the ability to derive human induced pluripotent stem cell (hiPSC) lines from any individual^10–13^ along with key advances in hESC/hiPSC differentiation into germ cells, affords now a major leap in the feasibility of IVG derivation and in the scope of their potential applications. Hence, in this paper we reappraise the ethical implications of IVG in the light of these momentous developments. Specifically, we develop an ethical framework, rooted in recent scientific evidence, well suited to support a morally robust experimental agenda that could enable the first-in-human use of IVG. We then apply this framework to the following objectives: (1) a clarification of the genetic parenting options afforded by IVG, along with their ethical underpinnings; (2) a defence of the use of IVG to remedy infertility, broadening their scope to same-sex couples; (3) an assessment of the most far-reaching implications of IVG for the expansion of reproductive autonomy. These include, first, the liberation of parenting roles from the constraints of biological generations in vivo, allowing multiple individuals to engage in genetic parenting together, thus blurring the distinction between biological and social generations. Second, we discuss the conflation of IVG with sequencing technology and its implications for the possibility that prospective parents may choose among a hitherto unprecedented number of potential children. In view of these perspectives, we argue that, contrary to the wide perception according to which society lags behind science, IVG may represent a salient instance of biotechnological ingenuity used to pursue social experimentation.

## In vitro gametogenesis

So far gametes of both sexes have been derived from mouse embryonic stem cells (ESCs) and induced pluripotent stem cell (iPSC) lines. These IVGs have been able to produce live offspring.^14–17^ Specifically, ESCs and iPSCs from mice were first differentiated in vitro into so-called epiblast-like cells, a transient state that recapitulates the formation of the pregastrulating epiblast in vivo, and which then yielded primordial germ cell-like cells (PGCLCs), the equivalent of the primordial germ cells (PGCs) that are set aside early in embryogenesis as the source of the future gametes. PGCLCs were then characterised at the molecular level and differentiated further into male germ cells (spermatozoa) by transplantation into testes of neonatal mice whose seminiferous tubules lacked germ cells.^15^ A slightly more complex version of the same approach was used to derive functional oocytes, again from murine ESC and iPSC lines. Here PGCLCs had to be first aggregated with somatic cells sourced from ovaries, a process that triggered key developmental transitions following which, upon transplantation, oocytes were obtained which could be then further matured in vitro and used in IVF to generate viable offspring.^16^ Most recently, the same authors further simplified the above protocol by inducing the expression of just three genes (coding for transcription factors (TF), ie regulatory proteins that themselves control the expression of many downstream genes) in epiblast-like cells, again yielding functional spermatozoa upon transplantation into mouse testes.^18^ The relevance of the latter findings lies first in the recapitulation, in the setting of germ cells, of similar successes achieved in reprogramming somatic cells into a panoply of unrelated cell types through a mere handful of TF, ushering in the current era of so-called ‘cell fate plug and play’.^19^ This permits the unravelling of the transcriptional logic that underlies germ cell fate and paves the way for rapid advances in the direct transdifferentiation of somatic cells into germ cells, possibly even bypassing eventually the intermediate attainment of various pluripotent states.

Second, the use of TF as a replacement of cytokines need not invite the scepticism that usually accompanies genetic manipulations with the fear that they would make such applications problematic in the human setting. For the simple reason that in many reprogramming paradigms the expression of TF is now routinely achieved through the delivery of messenger RNAs (the molecules transcribed from DNA that directly code for proteins) that do not integrate into the genome and thus avoid all issues—real or perceived—associated with the ‘scarring’ of the genome with transgenes. Indeed, the more precise our control of cell fate becomes, with the gradual replacement of elaborate culture media with the combinatorial expression of few key TF, the better position we will be in to obtain defined cell types amenable to the processes of standardisation and quality control that are necessary for their translation to the human setting.

As far as the human setting goes, instead, hESC and hiPSC have been differentiated in vitro into the equivalent of PGCs^20–22^ and very recently hiPSCs were also directly differentiated into haploid spermatogenic cells, thus demonstrating the accomplishment in vitro of defining milestones of human spermatogenesis in vivo.^23^ Human oocytes have until now not been derived from hESC or hiPSC, but progress in mice has not revealed any fundamental hurdle that should impede success also with human cells. We can thus summarise the current state of advance for the IVG field in terms of the validated results that were achieved so far, aligned to the sources of IVG. The sources include ESC and iPSC, in mice and humans. In turn, ESC lines can be derived either from IVF embryos or from somatic cell nuclear transfer embryos. In terms of validated results, we note the generation of viable offspring from either male or female IVG derived from murine ESC and iPSC. Viable offspring from gametes that were both generated in vitro has not yet been reported, and similarly no information is available about the longevity and general phenotype of mice generated by IVG. For human ESC and iPSC we can list instead the successful derivation of PGC and of haploid spermatocytes, thus recapitulating key in vivo milestones.

This current context has the following important implications for the framing of our bioethical analysis. First, taken together these results point to a substantial similarity, between mice and humans, of the key pathways that direct germ cell differentiation in vitro. Indeed, the very recent direct derivation of spermatocytes from hiPSC was achieved with protocols that had been previously defined for their murine counterpart.^23^ *Prima facie* we have therefore strong reasons to expect that human IVG would also prove equally functional in terms of live offspring generation. More than that, and certainly more importantly in terms of policy making and law enactment, these results give us a clear framework for the assessment of human IVG, since their functional murine counterparts were subjected to extensive transcriptomic and epigenomic profiles. This means that in the critical issue of how to assess the first-in-human use (see below), we will not need to start from a blank slate and will be able instead to advocate a rigorous pipeline to test human IVG functionality to the best of our current knowledge, buttressed by the undeniable strength of the murine in vivo results.

The gametes produced by IVG have been called *artificial gametes* and *synthetic gametes*. Sparrow, and Newson and Smajdor called them artificial gametes, while two of us have previously called them *synthetic gametes*.^2^ ^3^ ^24^ Here we propose the term of in vitro *generated gametes* (IVG) for two reasons. The first is that both terms *synthetic* and *artificial* no longer seem accurate and fair in the light of the scientific achievements we have summarised above. At least in mice, where IVGs have proven their full functionality, there is in fact no reason to regard them as ‘artificial’ or ‘synthetic’ simply because the way in which they are produced uses technology, much in the same way as we do not think of spectacles as providing ‘synthetic’ vision. After all, we do not think of Louis Brown as descending from an artificial or synthetic embryo, but simply from an in vitro fertilised one. Second, IVG is a more open-ended term that, by focusing on the process (ie, derivation in vitro) rather than on a purportedly essential feature of these cells, is more conducive to a pragmatic debate on their accommodation within our current palimpsest of reproductive options and parenting roles.

## The justification of human experiments using IVG

We now specifically explore the sources of moral justifications for creating a human being through a novel biotechnological procedure that has never been used in humans.

The first argument entails the comparison of the health of children born by IVG with the health of children born by other procedures that we consider morally unproblematic. For example, if we do not consider the results of natural reproduction to be morally problematic (with the possible exception of wrongful life cases), then other reproductive techniques with comparable results should *prima facie* be morally regarded in the same way.^25^ ^26^ This argument is grounded on the fact that moral consistency requires similar treatment of like cases.

One problem with this argument is that the only way to find out if the use of IVG for reproduction is as safe as natural or assisted reproduction would be by examining the results of a large number of human pregnancies achieved by IVG and compare them with the results of current pregnancies. This course of action might seem to involve an *impasse* because for the production of children through IVG to be regarded as morally unproblematic we need to know in advance, inter alia, how healthy IVG-generated children would be. The only option is thus to undertake extensive experiments in animals, always under appropriate ethical review, in order to assure that the beings born from IVG are comparably healthy to those born naturally. In parallel, careful studies should be initiated to monitor to great depth the development of IVG-derived human embryos in vitro for as long as it is legal (up to 14 days in many jurisdictions). Importantly, these studies should harness the full power of current sequencing techniques to probe, in single blastomeres sourced from these embryos, genomic and transcriptomic integrity vis-a-vis those of classic IVF embryos, a possibility that is already well within reach (see below). If the results from those experiments were favourable then we could think of using IVG for human reproduction on the basis of a solid scientific and ethical legitimation as far as safety is concerned. However, we would still be not completely sure of the long-term health of IVG-derived humans without undertaking the required experiment. Nevertheless, we believe that two lines of reasoning will justify the first-in-human application of IVG.

The first is that, as noted above, we now have a solid analytical pipeline to assess the developmental competence of human IVG on the basis of what we have learned from the extensive transcriptomic and epigenomic characterisation of their murine, functionally validated counterpart. More, with the most recent findings on the equivalence of murine and human IVG developmental milestones in vitro, it is fair to expect that by the time the prospect of IVG for human reproduction is considered, we will have a grid of markers and assays to prospectively isolate the IVGs that are most likely to result in viable healthy offspring. And it is fair to note that this level of scrutiny is not even comparable with the one that accompanied the first-in-human application of IVF.

At the time of Edwards and Steptoe's pioneering attempts^27^ there was neither the knowledge nor the technology to probe human IVF embryos at a similar level of molecular detail. Indeed if IVF had been subjected to the same scrutiny expected today for new medical procedures, it would have never come of age. Clearly, we are not advocating loosening today's rigour through the lens of the past. Yet, it is worth reminding ourselves that uncertainty is the defining feature of knowledge-intensive societies and applies, quite obviously, to any procedure contemplated in humans for the first time. If impractically high precautionary thresholds were decisive we would not have vaccines, nor IVF, nor any other advance. Nothing is entirely safe. We have to decide what's ‘safe enough’ given the balance of risks and benefits. Sometimes this decision must be left to those who wish to use the procedure and on whom the risk falls (given the conditions that we explore below). It is true that reproductive risks also fall on potential offspring. But that is true of all reproduction, and yet we do not ban it. Indeed, it is important to realise that we are already deeply engaged in a mass experiment on the quality of our gametes. This is particularly true for men, for whom ample evidence indicates that older age lowers the quality of gametes (most likely through the accumulation of DNA damage), with a clear correlation between older father's age and increased risk for several neuropsychiatric disorders like schizophrenia.^28^ Thus, allowing relatively older men to reproduce ‘naturally’ is already now causing effects that in the case of IVG could be brought up only hypothetically, namely the possibility that they result in deficits (like the neuropsychiatric ones that arise in early adulthood) that no mouse experiment or molecular profile on in vitro embryos could ever predict.

It should be clear that we are *not* saying that given that everything is in some sense *risky*, in reproductive scenarios anything goes. What we are saying is that when taking a moral stance, regarding the regulation of risky behaviours, or indeed on any matter whatsoever, there are good reasons to value consistency. If we are risk averse, then the same degree of risk should, other things being equal, attract the same degree of aversion; and if we value freedom, constraints on liberty of comparable nature and degree should be equally condemned. Departures from this principle of consistency are not necessarily unjustifiable but they require explanation and justification. Absent the adequacy of these explanations or justifications as to why other things are not equal, consistency requires that we treat like cases alike. Thus, in principle, all reproductive practices (that are inherently risky) should be equally assessed no matter their nature. When it comes to regulation, we are well aware that moral consistency must indeed take into account the processes as well as the consequences of actions. Thus, although it is manifestly inconsistent to value differently the creation of a child with disability depending on whether it happens in a laboratory or in a bedroom, it may well be reasonable to treat the two contexts differently. For example because of the importance attached to sexual freedom as well as to procreative liberty and to ancient and established liberties when compared with more recent claims. Consistency will however always be an important factor in assessing the comparative weight of these different features of explanation and the justification of departures from the principle of treating apparently like cases alike.

A second justification of the attempt to achieve a human pregnancy with IVG involves reminders about the role of uncertainty in decision making and uses the non-identity problem identified by Derek Parfit. Let's suppose that a woman takes an autonomous decision and gives her fully informed consent for the implantation of an embryo that was produced with IVG. If all steps of embryo culture following IVF with IVG proceeded normally (on the basis of the standards outlined above, including most likely the genomic and transcriptomic analysis of a single blastomere), then one could proceed to implantation. And if during any stage of the pregnancy any abnormalities were detected in development then, as in any other pregnancy, the pregnant woman should be informed of them and be asked if she wants to terminate it or carry it on.^25^ This is what happens, or should happen, when such features are detected in any pregnancy however initiated. Now, the non-identity problem tells us that if such pregnancy is carried to term there appears to be only one case in which the child born could have been wronged by the mere fact of her existence. This is when she has a life that it is not worth living. This conclusion is reached because reproductive choices, no matter how natural or technologically aided, are not bad overall for anyone that is brought into existence on condition that their life is on the whole worth living.^26^ It should be clear that the only other option for any created children would be never to have existed at all.

One issue that has always been present when dealing with the non-identity problem is that there appears to be something odd with the conclusion that as long as reproductive choices do not end in wrongful lives, no harm has been done. For example, if a women contracted a disease and then she decided to become pregnant, even though she knows that by having a child while sick any resulting child will have a mild cognitive impairment, we could not claim that she has harmed her child because the only other option for that child would have been to never have existed, and it is in that child's *interests* to exist even harmed in the ways indicated.

Parfit invites us to consider:

*The 14-Year-Old Girl.* This girl chooses to have a child. Because she is so young, she gives her child a bad start in life. Though this will have bad effects throughout the child's life, his life will, predictably, be worth living. If this girl had waited for several years, she would have had a different child, to whom she would have given a better start in life.^29^

This is analogous to the case of the IVG mother considered above who chooses IVG rather than other forms of reproduction. In both cases, two courses of action are open to the prospective mother. In criticising these women's pursuit of the first option available (ie, conception at 14 years or IVG) people might claim that each mother's decisions will probably be worse for *her child*.^30^ However, as Parfit has pointed out, while people can make this claim about the decisions taken it does not *explain* what they believe is objectionable about them. It fails to explain this because neither decision can be worse for the particular children born*; the alternative for both of them was to never to have existed at all*. If the 14-year-old waits to conceive, a completely different child will be born. Likewise, if the woman chooses not to use IVG and instead conceives by natural procreative means, or other assisted reproduction technologies, the child born will be a completely different one. Thus claims about the ethics of pursuing the first option in both of these cases cannot be claims about harm to *these* children. It is better for *these* children that they live when the relevant alternative was never to have lived.

This does not of course mean that parents do not have moral reasons to have different children in better circumstances, rather it means that the reasons for not procreating, or not permitting or assisting procreation, are not that such a course is in the *interests* of ‘the child who may be born’.^i^ The moral reasons for preferring to have children in better rather than worse circumstances, where this means that the children who come into being will be different, can be of various sorts but these would have to amount to a claim that the outcome would be better overall. Or, perhaps that the world that would be created by the decision would be better in some defensible sense than the alternative world. However, while there may be reasons for moral criticism of the choices made by either Parfit's 14–year-old girl, or by the IVG mother, it is important to be clear about two things. First that such criticism cannot appeal to the interests of ‘the child who may be born’ for the reasons just outlined. Second, we should remember that suboptimal parenting may be considered to be standard in human affairs and we have no reason to insist always that medically or technologically assisted parenting should always and only be required to be optimal.

Consider that poverty is one of the most reliable predictors of bad outcomes for children and indeed for all who live in poverty.^31^ However we do not judge that for these reasons the poor should either be prevented from reproducing or be denied medical or technological assistance with procreation. The non-identity problem is one source of justification for such a position. Others include reluctance to discriminate against people in poverty, not least in the provision of medical assistance with something so important as procreation. The therapy of choice here is, as in so many other cases, not further impositions on people in poverty, but more radical steps to eradicate extreme poverty and its effects.

## A typology of uses for IVG in infertility

The development of IVG could have a significant impact on human reproduction as a watershed remedy for infertility. It is descriptively useful, on scientific and bioethical grounds, to distinguish the use of IVG to restore ‘natural’ fertility in individuals or couples from their application to expand human fertility beyond current limits. We do not take this distinction to imply any normative primacy on the side of the ‘natural’, but just as a discursive tool to frame the topic and bring to salience the relevant similarities and differences among the various options. The envisaged application of IVG has so far been the restoration of fertility in individuals or couples who could be normally expected to be fertile save for inherited or acquired deficits in the reproductive proficiency typical for their age, sex and health. As Sparrow rightly notices, this includes:(1) post-puberty men who are unable to produce viable sperm; (2) women who have undergone premature menopause; and (3) those who have lost their gonads due to injury or had them removed in the course of cancer treatment.^24^ We add here a fourth category, that has been overlooked until now, namely that of men and women who have been subjected to biological involuntary sterilisation (BIS) and want to become genetic parents. BIS refers to any procedure, instituted in violation of a subject's autonomy, that impairs temporally or permanently the subject's capacity to reproduce. Although one usually associates BIS with the Nazi's eugenics programmes of the first half of the 20th century it is less well known that from the 1990s to the present day egregious cases of involuntary sterilisation have still taken place in different regions of the world, including Peru,^32^ Namibia,^33^ Uzbekistan,^34^ Slovakia^35^ and even Sweden,^36^ though in the latter case this was not involuntary sterilisation tout court*,* but rather the mandatory sterilisation required by the state from individuals who underwent sex reassignment surgery and wished to have the new gender recognised by the authorities.

For all these individuals the use of IVG, with or without surrogacy, could become the only option to have genetically related kin. Beyond them, however, the feature of IVG that could revolutionise human reproduction, if proven to be effective and safe within the analytical framework we advocated above, will be the expansion of reproductive options to beings, individuals or couples that are not currently expected to be fertile, who can be grouped into six categories on the basis of different scientific and ethical implications.

The first concerns cell lines; and here we see little if any scope in their use for human reproduction (save for their transient use in the expansion of reproductive autonomy to multiplex parenting that we discuss below). The only request could come from individuals or couples who might prefer to resort to IVG derived from existing cell lines (rather than from in vivo or in vitro generated gametes sourced from an existing individual), presumably on the basis of their perceived genetic superiority. Scientific and ethical reasons suffice to discourage such use, for fertile individuals who only want to have a healthy genetically related child. First, prolonged culture (even in cell lines that are not obviously transformed such as ESC or iPSC) poses greater risk of DNA damage. Second, the recurrent use of few lines for reproduction, just as in the case of too frequent sperm donations, is possibly problematic for the risk of inadvertent interbreeding among descendants. Third, in the hypothetical case of using cell lines that are not sourced from an individual who actually ever existed (ie, ESC as opposed to iPSC), whether or not the interest of the offspring in relating, even if just potentially, to a biological parent with an actual biography, should trump individuals’ or couples’ entitlement to pursue their reproductive autonomy with ESC-derived IVG is an open question beyond the scope of this paper.

The second group concerns embryos, fetuses and children. Also in this case we regard this possibility as a mere scholarly hypothesis with little if any scope in practice (save for the transient use of embryos in multiplex parenting discussed below). The ethical assessment of this group depends on whether the embryos and fetuses will be carried to term. If they are not going to be carried to term, the problem of the offspring's interest in relating to a biological parent with an actual biography is the same as outlined above for ESC lines. On the other hand when embryos and fetuses are going to be carried to term, a possible argument against reproduction with IVG derived from them, as well as from children, is the violation of their reproductive autonomy, given their inability to decide whether and how to become a biological parent.

The third group concerns deceased individuals, and does not pose specific issues other than those that have already been successfully addressed for posthumous fatherhood.^37^ ^38^

The fourth group concerns postmenopausal women, and here as well we believe that the ethical framework that currently supports postmenopausal pregnancies with donor gametes could well be invoked to support the use of IVG in the same setting.

The fifth group concerns single individuals, who may wish to reproduce without partner and without resorting to gamete donation, a scenario that would become relevant for policy only if it were effective and safe (see below) to generate gametes of both sexes from either men or women. This self-breeding setting would be different from reproductive cloning in one critical aspect, namely that reassortment of alleles at meiosis could increase the chance that deleterious heterozygous mutations are brought to homozygosity in the offspring, even more likely than what happens between siblings or first-degree cousins. The issue of whether or not such choices should be left open, rather in the way that we do not prevent people who will inevitably pass on adverse genetic conditions from reproducing, is a question that we have partially addressed above when talking about moral consistency, but it needs to be more deeply explored. Whether reproducing ‘by oneself’ in this way is a value that requires protection at the costs we have noted seems however unlikely.

## Same-sex couples

Finally, the sixth group is the one for which we see greatest scope in practice and more far-reaching transforming implications, and hence the one that is going to receive most attention: that of same-sex couples who would be able to have children as closely genetically related to them as those produced by couples through sexual reproduction.^1^

Right now there are a variety of options for same-sex couples that want to become parents: adoption, gamete donation plus surrogacy, gamete donation without surrogacy, embryo adoption plus surrogacy and embryo adoption without surrogacy. What all these options have in common is that one, or both, of the future parents will provide less genome and epigenome content than in sexual reproduction and perhaps none at all. This means that in the end both members of the couple will become parenting parents but only one, or none, will be genetic parents. What is revolutionary about IVG is that by means of in vitro *gametogenesis* it might be possible to derive functional gametes of both sexes from either male or female hiPSC, thus allowing both members of a same-sex couple to become genetic parents. Specifically, the fact that sperm and oocytes were derived from male ESC^39–41^ suggests that eventually same-sex male couples could have a child that is genetically related to both of them via IVG, IVF and surrogacy. The same has not yet been achieved with female ESC, since so far only oocytes were derived from them, yet the creation of a mouse with the DNA of two female mice makes us aware of the theoretical possibility of a child that could be genetically related to both mothers in the same *ratio* as a in current reproduction.^2^ ^24^ ^42^ We think that all competent caring people have the same legitimate interest in becoming parenting parents, just as those who happen to be able to reproduce sexually, and also that they have a legitimate interest in wanting to be the genetic parents of their children. There is nothing morally wrong with same-sex competent caring people using IVG for satisfying their legitimate interests in becoming genetic parents of their children.

## IVG and multiplex genetic parenting

Beyond same-sex genetic parenting, the most paradigm-shifting application of IVG could be a radical expansion of reproductive autonomy that allowed more than two persons to engage simultaneously in genetic parenting. While the notion of the couple (and coupling) currently dominates sentimental relationships worldwide, several societies still practice or tolerate other bonding models through various forms of polygamy. Moreover, while also in the West more or less famous ‘private’ trios have always abounded amidst varying degrees of sanction, the legal tool of ‘civil union’ enabled recently the first public recognition of a three-way relationship between one man and two women.^43^ Currently, for three or more individuals who wanted to share genetic parenting, the only theoretical option would be through replacement of mitochondrial DNA (mtDNA), creating an embryo inheriting the nuclear genome from a man and a woman and the mtDNA from a second woman. In the UK this approach has been recommended by the Human Fertilisation and Embryology Authority as ethically sound for the prevention of devastating diseases due to mtDNA mutations and entails only a minimal mixing of genetic material. Indeed, precisely this feature has been invoked to dispel fears of three-way genetic parenting.^44^ ^45^ IVG could permit instead a much more substantive sharing of genetic kinship, through what is in essence a generational shortcut. Imagine that four people in a relationship want to parent a child while being all genetically related to her. IVG would enable the following scenario: first, two embryos would be generated from either couple through IVF with either naturally or in vitro generated gametes. hESC lines would be then established from both embryos and differentiated into IVG to be used in a second round of IVF. The resulting embryo would be genetically related to all four prospective parents, who would technically be the child's genetic grandparents. In light of the developments we have anticipated above, several variations are possible over this scheme, including trios and same-sex partnerships, though in the case of trios the extent of inbreeding would need to be dealt with on a par with that outlined above for self-reproducers.

If we find it morally unproblematic that people who cannot achieve natural reproduction rely on assisted reproduction to have genetically related kin then we find no reason why this should not hold also for non-couple partnerships for whom simultaneous genetic kinship is currently prevented, given that they will provide the necessary parenting and care for the resulting children. We are well aware that the genome is only one, albeit critical element in the inheritance and deployment of phenotypes, and two of us have argued at length against the fallacies of genetic determinism and against poor scientific journalism that promotes an unreflective salience of genes in the public sphere.^26^ ^37^ From an ethical standpoint, however, it is clear that short of granting moral primacy to the purported ‘natural’, no cogent argument allows the restriction of the option of genetic kinship only to specific categories of individuals or couples. And as far as the likely argument is concerned that such multiplex genetic parenting would be bad for the child, we could simply reiterate, at its most basic, that also the children born through this application of IVG would not be harmed by being brought into existence because the only other option would be never to have been at all (as we have previously discussed).

However, we find it more productive to ground the legitimacy of this reproductive option on a deeper assessment of the meaning of human genealogy. As one of us has argued elsewhere,^37^ the so-called ‘nuclear’ family that combines genetic inheritance with parental care is just one, and for that matter relatively recent configuration in the history of human relationships. From milk-mothering through adoption to surrogacy, from tribal upbringing to patchwork-families, parenting modes have diversified over a wide range of options in space and time (It should be noted that when talking about parenting we are indeed talking about parenting as a specific type of caring and not only about child caring, given that not all types of caring is parenting). In space, by being more or less directly engaged with the body of the offspring, from the inheritance of traits from biological parents through the epigenetic impact of milk mothers and surrogates to the brain wiring triggered by parents, and other carers, etc. In time, by being more or less directly related to the actual genetic generations, think only of the key role that grandparents or uncles have been playing in child rearing. In other words, the way we are has always resulted from the complex interplay of our genomic and epigenomic individuality,^46^ with the latter a crowded site of convergence for multiple parenting roles belonging to different generations. IVG would now allow the genome to be distributed and shared equally, bending the temporal necessity of genetic generations to the social and cultural preferences of our times. The in vitro compression of generational time appears thus like the most transforming feature of IVG derivation, and while Sparrow has highlighted its important implications for research on human genetics and development,^24^ we find its impact on human reproduction even more far-reaching. Indeed, by taming genetic kinship for parenting preferences, IVG may well be considered the most salient example in that coproduction of biotechnological pluralism, whereby normative commitments recruit biotechnological ingenuity to turn possible lifestyles, that although morally acceptable are not biotechnologically feasible, into actual living options.^47^

## IVG, sequencing and the generations to come

The final aspect of IVG that is particularly worth noting relates directly to its ability to provide a quantitatively unprecedented supply of human eggs and sperm. Shortage of eggs in particular has been the rate-limiting step for the development of large-scale in vitro human genetics. With IVG a couple that went through IVG plus IVF could generate a large number of embryos from which to select.^4^ The potentially paradigm-shifting impact of having large numbers of embryos from which to select comes into relief only if we consider, in parallel, the skyrocketing advances in sequencing, including its miniaturisation. Nowadays, preimplantation genetic diagnosis has a limited readout, because the single cell that is removed from the embryo can be probed only for a handful of genetic abnormalities. But in the research setting it has already proven possible to obtain whole genome sequencing information from single cells, including individual blastomeres of a human embryo.^48^ And it is already feasible to mine the transcriptomes of single cells, which again can also provide basic information about the underlying genome status.^49^ In short, with the exponential progress in sequencing efficiency and the attending computational analytical pipelines, along with the plunging costs of the various sequencing platforms that are competing in the market, it is very likely that in the near future individual blastomeres from several embryos will become amenable to high throughput sequencing. Akin to what is currently happening (and anticipated to happen on an even greater scale) to patients, research participants or consumers confronting gene test results, especially in the direct-to-consumer setting,^50^ parents will be exposed to the possibility of selecting from a wide variety of genetic features, whose consequences, however, will remain for the foreseeable future only very partially understood.

## Conclusion

In this paper we have developed an ethical framework, rooted in recent scientific evidence, to support a robust experimental pipeline that could enable the first-in-human use of IVG. In achieving this we have set out the genetic parenting options afforded by IVG, along with their ethical underpinnings; we have mounted a defence of the use of IVG to remedy infertility, broadening their scope to same-sex couples and have set forth the far reaching implications of IVG for the expansion of reproductive autonomy. Henceforth reproductive autonomy could involve the liberation of parenting roles from the constraints of biological generations in vivo, allowing multiple individuals to engage in genetic parenting together, thus blurring the distinction between biological and social generations. Finally prospective parents will be able to choose among a hitherto unimaginable variety of potential children. For these reasons, we have argued that biotechnological ingenuity could now be harnessed in the service of social experimentation thus reversing the usual pattern whereby social experimentation is perceived to follow slowly behind scientific progress.

## Footnotes

i This term of art was used in *The Human Fertilization and Embryology Act 1990.* Clause 13.5. of that Act states: “A woman shall not be provided with treatment services unless account has been taken of the welfare of the child who may be born as a result of the treatment (including the need of that child for a father), and of any other child who may be affected by the birth.” See: Harris J. The welfare of the child. Health Care Anal. 2000;8(1):27–34.1–8.
